# Interaction Structures in Psychodynamic Psychotherapy for Adolescents

**DOI:** 10.3390/ijerph182413007

**Published:** 2021-12-09

**Authors:** Barış Can, Sibel Halfon

**Affiliations:** Psychology Department, Faculty of Social Sciences and Humanities, Istanbul Bilgi University, Santralistanbul Eski Silahtarağa Elektrik Santralı Kazım Karabekir Cad. No: 2/13, Istanbul 34060, Turkey; baris.can02@bilgiedu.net

**Keywords:** adolescent psychodynamic psychotherapy, interaction structures, Adolescent Psychotherapy Q-Set

## Abstract

Despite advances in psychotherapy research showing an evidence-base for psychodynamic psychotherapy (PDT) in adolescents, developmentally specific treatment characteristics are under-researched. We aimed to identify interaction structures (IS: reciprocal patterns of in-session interactions involving therapist interventions, patient behaviors, and the therapeutic relationship) and assess associations between IS and outcome. The study cohort comprised 43 adolescents (*M*_age_ = 13.02 years) with nonclinical, internalizing, and comorbid internalizing–externalizing problems in PDT. A total of 123 sessions from different treatment phases were rated based on the Adolescent Psychotherapy Q-Set (APQ). Outcome was assessed with the Brief Problem Monitor-Youth (BPM-Y) administered repeatedly over the treatment course. Principal component analysis of APQ items resulted in five IS, named “Negative Therapeutic Alliance”, “Demanding Patient, Accommodating Therapist”, “Emotionally Distant Resistant Patient”, “Inexpressive Patient, Inviting Therapist”, and “Exploratory Psychodynamic Technique” (EPT). Multilevel modeling analyses with Bayesian Markov chain Monte Carlo (MCMC) estimations indicated a two-way interaction effect between EPT and problem levels at baseline such that patients with lower problems at baseline showed good outcome in the context of EPT, whereas an inverse relationship was found for patients with higher problems. Findings provide empirical evidence for characteristic components of PDT for adolescents and preliminary answers about who benefits from psychodynamic techniques.

## 1. Introduction

Recent reviews on the evidence base of psychodynamic psychotherapy (PDT) with children and adolescents have shown support for the efficacy of PDT for a wide range of mental disorders [1,2,3,4,5]. However, knowledge is limited on the core treatment processes that may answer how PDT with adolescents works and on specific treatment markers which can identify who benefits most from this modality [6]. This knowledge gap is due (at least in part) to a lack of appropriate measures that can meaningfully capture the microprocesses taking place within sessions. The Adolescent Psychotherapy Q-set (APQ) [7] (which is derived from the Psychotherapy Process Q-set (PQS) [8] for adult psychotherapy and the Child Psychotherapy Q-set (CPQ) [9] for child therapy) provide opportunities to closely study many core mechanisms that take place within sessions. The APQ is composed of 100 items that describe the adolescent’s experiences, the therapist’s attitudes, interventions, and the nature of their interaction. The APQ incorporates items that reflect the core issues of the adolescence period [10], which enables researchers to identify rudimentary, recurring, conscious, and unconscious relational patterns that emerge in treatment between the therapist and patient dyad (i.e., interaction structures (IS)) [8]. IS have been used to better understand the nature of the therapeutic process by analyzing PDT sessions with adults and children (with the CPQ and PQS, respectively) using single cases, e.g., [11,12,13] and cross-sectional studies, e.g., [14,15]. However, very few studies have extracted IS in PDT with adolescents, e.g., [10] and none of these studies assessed associations with the outcome.

The purpose of this study is to extend the methodology and guiding question of Q-set studies to PDT in adolescents. Our first aim was to attain better understanding of the PDT process for adolescents with nonclinical, internalizing, and comorbid internalizing–externalizing problems through the extraction of emergent IS in the course of their therapies. The second aim was to investigate whether the identified IS can predict the outcome.

### 1.1. Evidence Base and IS for PDT in Adolescents

While the evidence base for adolescent PDT grows, recent studies have underscored the specific predictors and differential therapeutic responses to child–adolescent PDT [1,3,4]. Emotional and internalizing problems respond better to psychotherapy than those with disruptive disorders and adolescents with more severe psychopathology [14,15,16,17]. In the largest and best conducted RCT using PDT in adolescents (the IMPACT study) [18,19], those with higher symptoms showed “halted improvement” with faster early recovery, but long-term improvement was not observed.

These different trajectories of change suggest that there might be separate mechanisms in action. Studies using the APQ have extracted specific IS unique to PDT in adolescents. The emergent IS mostly reflected the quality of the therapeutic relationship and the specific relational challenges that may arise with this population. Calderon and colleagues [20] used a relatively equal number of Short-Term Psychodynamic Psychotherapy (STPP) and Cognitive Behavioral Therapy (CBT) sessions from early/mid and mid/late stages of therapy from a group of depressed adolescents. They found characteristics of a strong working relationship as well as a difficult working relationship if there was limited engagement from the adolescent and difficulty making progress [20]. Grossfeld et al. (2019) [21] applied the APQ in a single case design to the therapy of a depressed adolescent with borderline personality disorder (BPD). Their emergent IS grouped Q-set items around therapy progress (whether fluent or stuck), along with therapeutic processes involving emotions, such as working with painful emotions and the patient’s expression of anger. Di Lorenzo and Maggiolini (2019) [22] extracted IS based on experts’ ratings of Q-set items with regard to a characteristic PDT session in adolescents. Their IS showed a continuum of expressive (e.g., interpretations) to supportive (e.g., offering guidance) psychodynamic techniques that were employed by the therapists and signs of alliance ruptures. Those studies indicated that several techniques (supportive to expressive) and therapeutic–alliance characteristics take place in PDT sessions involving adolescents. However, none of those studies investigated associations between IS and outcome to bring together core process and outcome factors.

The only studies that investigated associations between IS and outcome in cross-sectional samples were conducted with children and adults via the use of CPQ and the PQS. Halfon et al. (2020) [23] and Halfon (2021) [24] found that, even though the psychodynamic technique IS predicted the outcome, this was moderated by problem type and severity, with children showing externalizing and comorbid psychopathology benefiting more from client-centered and supportive approaches. Similar findings were found with adults. Even though psychodynamic techniques were (in general) associated with a good outcome, patients with low and more severe levels of psychological distress benefited from different techniques, such that supportive techniques predicted a good outcome for those with high distress severity at baseline whereas expressive techniques were associated with improvement for those with low distress severity at baseline [25].

### 1.2. Aims of the Current Study

Our first aim was to investigate the specific IS that emerge in PDT with a group of adolescents with nonclinical, internalizing and comorbid internalizing–externalizing problems. Studies in adolescent PDT have consistently identified IS associated with different components of the therapeutic alliance (i.e., strong working relationship and difficult working relationship) [20] and of the psychodynamic technique (i.e., supportive, explorative, and expressive) [20,22]. Therefore, we expected to extract IS associated with these constructs. However, due to limited literature, we were not able to set forth specific hypotheses regarding other IS that would emerge.

The second aim was to assess associations between the IS and outcome. Studies using PQS and CPQ found that even though the psychodynamic technique IS could predict the outcome, this was moderated by symptom severity [24,25]. Therefore, we aimed to test the effect of problem levels at baseline on our second aim. The specific hypotheses were that: (1) we would be able to identify distinct and conceptually sound IS summarizing the psychotherapy process; (2) we would extract IS associated with the therapeutic alliance and psychodynamic technique; (3) the IS would predict the outcome; (4) the problem levels of patients at baseline would moderate the association between the psychodynamic technique IS and outcome such that patients who start treatment with lower problem levels would benefit more from psychodynamic techniques.

## 2. Materials and Methods

### 2.1. Study Design

The study protocol was approved by the Ethics Committee of Istanbul Bilgi University (Ethical Approval Code 2015-400-24-11, Istanbul, Turkey). Before starting treatment, patients and their parents were informed extensively about the research procedures. Parents and adolescents provided written consent for us to use their data, which involved administered questionnaires and audio/video recordings of their sessions.

Data were collected at the Psychotherapy Laboratory of Istanbul Bilgi University within the framework of a research program that seeks to investigate the process and outcome of PDT in children and adolescents (for details, see [24]). This study followed a naturalistic process–outcome design. The sessions took place between Fall 2016 and Spring 2019. Data were collected from a group of adolescents who applied for services at Istanbul Bilgi University Psychological Counseling Center (BUPCC), a mental health training clinic which provides low-cost PDT in the outpatient setting.

### 2.2. Participants

The referrals for adolescents to the BUPCC were made by the patients’ parents or by professionals in mental health, medical, or child welfare services. Upon referral to the clinic, parents and adolescents were screened by a clinical psychologist with over 10 years of clinical experience trained in developmental psychopathology and psychiatric-interviewing techniques. In this way, we determined whether the patients fit the inclusion criteria for enrollment.

Adolescents (11–17 years) were included in our study. The exclusion criteria were patients with psychotic symptoms, patients who abused drugs, patients who carried immediate or significant suicidal risks, or patients with primary eating disorders. These patients were referred to the relevant services for treatment.

The study cohort comprised 43 adolescents aged 11–17 years (mean (*M*)_age_ = 13.02, standard deviation (*SD*) = 1.85). Approximately four-fifths of patients were in their early adolescence (11–14 years), with the remaining in mid-adolescence (15–17 years). The percentage of females was 46.5%. All patients resided in a metropolitan urban setting and arose from families of low-to-middle socioeconomic status. The reasons for referral were heterogeneous: depression and anxiety (37%), aggressive behaviors (23%), social and academic difficulties (30%), and adjustment difficulties (10%). The study cohort was relatively homogeneous in terms of problem levels, and 70% of children were at “clinical” levels of functioning (*M* Total Problems *T*-score = 60.55, *SD* = 10.76) on the Youth Self-Report (YSR) [26,27], in which a T-score > 60 indicates clinical functioning. Additionally, 41% of patients were discrete internalizers and 29% were comorbid internalizers–externalizers; no discrete externalizers were in our cohort.

### 2.3. Therapists

The therapists were 29 students in clinical psychology at the level of an advanced master’s degree. Most (82.8%) were women aged between 23 and 37 years (*M* = 25.76, *SD* = 3.20). Each therapist had been educated in the theoretical background of PDT for 2 years during classes, supervisions, and case seminars for a master’s degree. All therapists had 1–2 years of supervised experience in psychotherapy. Each clinician received ≥4 h of supervision per week (1 h individually and 3 h in a regular supervision group) from psychotherapists with >10 years of clinical experience in PDT.

### 2.4. Treatment Adherence

The standard treatment provided at BUPCC is PDT that broadly follows the practice parameters outlined by Kernberg et al. (2012) [28]. In summary, the therapist encourages the young person to initiate conversation about personally meaningful events, experiences, and significant issues. Play is also incorporated into the therapy process for some cases (especially younger adolescents) to help them express and reflect on their perceptions, feelings, and thoughts. The therapist places central importance on adolescents’ recurrent emotional and interpersonal experiences and on unconscious functioning, including wishes, dreams and fantasies, along with early memories. The therapist and adolescent work together to reflect on the symbolic meaning of behaviors and experiences, and try to uncover unconscious conflicts. The ways in which the adolescent avoids difficult experiences and contradictory feelings are explored, and the defenses against these experiences are interpreted gradually. The therapist also draws attention to the therapy relationship to highlight the adolescent’s emotional and interpersonal patterns that find reflection in transference–countertransference dynamics. Collateral parental sessions take place once a month with or without participation of the adolescent, depending on the individualized therapy frame. In the parent sessions, the parents and therapist reflect on the parents’ and adolescent’s issues to help the parents understand the mental states of the adolescent and their relationship.

The treatment process began with a standard assessment which lasted ~4 sessions. In this standard assessment, a clinical interview was conducted with the parents and the adolescent to learn about the history of the presenting problem, as well as the developmental history and family background and relationships of the adolescent. Then, the therapist presented a clinical formulation and treatment plan. The standard treatment plan at the clinic involved a once-weekly therapy session of 50 min with the adolescent, along with once-monthly parent sessions. Treatments were open-ended in length and determined based on progress toward goals, life changes, and the decisions made by the family of the patient. The duration of treatments varied among our 43 participants, with the mean number of sessions being 24.49 (*SD* = 13.13).

“Ideal” prototypes for APQ are lacking, so adherence to the psychodynamic process was checked by comparing the most and least characteristic Q-set items with the PDT prototype created by Bychkova et al. (2011) [29]. According to the profile of the sessions, the therapists were nonjudgmentally inquisitive, asked, and encouraged the patients to talk about and elaborate upon their experiences in an effort to clarify and make sense of them. Along with exploration of interpersonal relationships, the focus of the therapy was on the internal world of patients, and especially on the “here and now”. Furthermore, the therapists refrained from more structured techniques, such as psychoeducation, problem-solving, discussion of tasks, and suggesting alternative ways of relating to others outside therapy. They also tended to limit use of supportive and structured techniques, such as guidance or direct reassurance of patients. The patients initiated and elaborated on pertinent topics. Patients felt understood, in congruence with the therapists’ tendency not to make definite statements about the patients’ mental worlds. Comparison of the most characteristic items with the prototypes in the study by Bychkova and colleagues (2011) [29] revealed that the current sessions shared eight items (3, 9, 18, 63, 65, 96, 97) out of the 10 items of the PDT prototype, which indicated strong adherence to the PDT model.

### 2.5. Measures

The Background Information Form was used to acquire demographic information, such as age, education, and socioeconomic status.

The Adolescent Psychotherapy Process Q-Set (APQ) [7] is used to analyze the psychotherapeutic process among patients aged 11–18 years. This instrument consists of 100 items. It contains statements that describe a feature of the treatment process corresponding to the (a) adolescent’s attitudes (e.g., item 13: “Young person is animated or excited”); (b) therapist’s actions and attitudes (e.g., item 17: “The therapist actively structures the session”); and (c) nature of the patient–therapist interaction (e.g., item 98: “The therapy relationship is a focus of discussion”). The raters initially watch/listen to the recording of a therapy session. Then, they use a forced-choice technique to Q-sort the items from most uncharacteristic (pile 1) to most characteristic (pile 9), aiming to place a fixed number of items in each category to approximate to a normal distribution.

APQ studies have shown adequate interrater reliability among coders e.g., [10,20]. The convergent and discriminant validity of the APQ was established by first conducting a Q-factor analysis of STPP and CBT sessions from the IMPACT study [13]. Psychodynamic and CBT factors diverged meaningfully between the STPP and CBT sessions and correlated significantly with other similar scales. The APQ was also able to differentiate key features of four theoretical schools that fall under PDT [22].

The coders in the present study were five research assistants at the level of a master’s degree trained by the second author. They were independent from the treating clinicians or cases, and were blinded to study objectives. They initially Q-sorted practice videos to reach an intraclass correlation (ICC) of 0.70. Afterward, pairs of coders coded the sessions independently and reached a satisfactory ICC of 0.70–0.94 (*M* ICC = 0.80). Then, the two sets of independent ratings were composited by taking their mean value.

The Youth Self-Report (YSR) [26] is used widely to assess a large array of emotional, social, and behavioral problems in young people (11–18 years). A total of 112 problem behavior items that constitute the main body of the YSR ask the young person to rate each item (e.g., “I am shy and timid”, “I am stubborn”, “I like to help others”) within a three-point scale (0 = “not true”, 1 = “somewhat or sometimes true”, and 2 = “very true or often true”). The outcomes are categorized under internalizing (e.g., anxious/depressed), externalizing (e.g., rule-breaking behavior), and Total Problems. The YSR has good internal consistency (*α* = 0.83) and acceptable test–retest reliability (*r* = 0.79) [27]. Erol and Şimşek (2000) [30] adapted the Turkish version of the YSR, which has shown excellent internal consistency (*α* = 0.89) and good test–retest reliability (*r* = 0.79) for the Total Problems scale. The YSR Total Problems scale in the current study had excellent internal consistency (*α* = 0.95).

The Brief Problems Monitor-Youth (BPM-Y) consists of 19 items taken from the more comprehensive YSR via item response theory and factor analysis [27]. BPM-Y items utilize a three-point scale (0 = “not true”, 1 = “sometimes or somewhat true”, 2 = “very true or often true”) and assess problem behaviors under internalizing (e.g., “I am too fearful and anxious”), attention problems (e.g., “I am inattentive and easily distracted”), and externalizing problems (e.g., “I argue a lot”). The overall Total Problems score has a high level of internal consistency (*α* = 0.86) and test–retest reliability (*r* = 0.88), along with criterion validity [31]. An excellent degree of internal consistency was identified for the BPM-Y Total Problems scale in the current study (*α* = 0.91).

### 2.6. Procedures

The YSR was administered to adolescents during the intake and at the final session of their treatments. All adolescents were also assessed on problem levels at regular intervals (every tenth session in treatment) with the BPM-Y. All psychotherapy sessions were videotaped and transcribed. For in-session APQ codings, the session on which the BPM-Y was completed was chosen by the principal investigator. The sessions were then arranged in random order, and coded independently. A total of 123 sessions from every tenth session in each adolescent’s treatment was coded.

### 2.7. Data–Analytic Strategy

To test hypothesis 1 and hypothesis 2, a principal component analysis with direct oblimin rotation was conducted to identify IS using SPSS 26 [32] (IBM, Armonk, NY, USA). The factors were determined according to the cumulative variability explained and their interpretability.

To test hypothesis 3 and hypothesis 4, due to our data structure whereby sessions (*N* = 123) were nested within patients (*N* = 43) who were nested within therapists (*N* = 29), we used a multilevel modeling (MLM) approach based on Bayesian analyses with Markov chain Monte Carlo (MCMC) estimations employing MLwiN 3.05 (Bristol University, Bristol, UK) [33]. In the case of complex data with small sample sizes and a high number of predictors and cross-level interactions, MCMC estimations have marked benefits compared to full maximum likelihood (FML) or restricted maximum likelihood (REML) models to produce unbiased estimates [34]. MCMC is a simulation-based approach which uses an iterative fitting procedure that yields posterior distributions using initial starting estimates. Maximum likelihood values are usually used as starting estimates to derive more reliable final estimates. Prospective diagnostics in MCMC models indicate the number of simulations needed to get stable distributions as well as an estimate of Effective Sample Size (ESS), which is the sample size required to achieve the same level of precision if the sample was random. An ESS of 500 is sufficient for most parameters of interest [35]. 

The first step of the analyses was to form “empty” MLM models with BPM-Y Total Problems as the dependent variable. The therapist-level ICC for BPM-Y Total Problems was 0.00, n.s., indicating that no significant variability at the therapist-level. In contrast, the patient-level ICC was 0.67, *p* < 0.01; therefore, a two-level model was chosen.

Afterward, two separate models were developed, one to measure change in problem levels (i.e., BPM-Y Total Problems) and another to measure the effects of IS on outcome. We initially estimated parameters by marginal quasi-likelihood methods (RIGLS), which were used as “priors” for Markov chain MCMC models. Ninety-five percent Credible Intervals (CrI) were derived from the 2.5 and 97.5 percentile values of the MCMC estimations. An initial burn-in phase of 500 followed by 5000 iterations were specified. The trace plots for each parameter were visually inspected in order to assess whether the model reached equilibrium distribution.

## 3. Results

### 3.1. Extraction of IS

A principal component analysis with direct oblimin rotation resulted in five IS that accounted for 40% of the shared variance (Appendix A: Table A1).

IS 1 accounted for 16.01% of the variance (*α* = 0.88) and was labeled “Negative Therapeutic Alliance”. Positive loading items included the young person’s negative bond and noncompliance with therapeutic tasks, such as not initiating or elaborating on topics (items 15, 30), acting provocatively during sessions (item 20), greater episodes of silence (item 12), and expression of (verbally and/or nonverbally) a distrustful or dismissive attitude toward the therapist (items 1, 42, 44). Negative loading items represented the young person’s lack of investment in the therapy process (items 73r, 95r), lifelessness, and somber mood (items 13r, 74r). The IS was also marked by a decrease in the young person’s mentalization capacity, with lowered interest in the minds of others (item 23r), lack of agency (items 28r), and inability to connect behaviors and mental states (item 24r).

IS 2 accounted for 8.67% of the variance (*α* = 0.83) and was labeled “Demanding Patient, Accommodating Therapist”. Positive loading items indicated the patient’s attempts to control sessions (item 87), to pressure the therapist to meet his/her demands (item 83), and needs for approval, affection, and sympathy (item 78). The therapist’s response was remaining thoughtful (item 37), refraining from taking a position (item 93), but still accommodating the adolescent’s strong emotions (item 47), even though the therapist could also make definite comments if faced with the adolescent’s demands (item 89). Negative loading items represented the adolescent’s dependency (item 29r), disorganization in expression (item 54r), and the therapist exploring the adolescent’s concerns in lieu of taking a psychoeducational stance (item 33r) or offering a different perspective (item 80r).

IS 3 accounted for 6.98% of the variance (*α* = 0.80) and was named “Emotionally Distant Resistant Patient”. Positive loading items indicated that the young person resisted the therapist’s attempts to explore his/her internal world (item 58), had difficulty maintaining attention (item 67), and was distant from his/her feelings. Negative loading items indicated that negative emotions, such as remorse (item 22r), inadequacy (item 55r), vulnerability (item 8r), sadness (item 94r), rejection, abandonment (item 41r), and troublesome affect (item 26r), were disavowed. The adolescent was less likely to achieve a new understanding (item 32r) and the therapist responded by drawing attention to nonverbal (item 2) rather than explicit interventions to make sense of experiences (item 9r).

IS 4 accounted for 6.61% of the variance (*α* = 0.75) and was named “Shy Patient, Inviting Therapist”. Positive loading items mostly described a young person acting in a shy or self-conscious manner (item 61) and the therapist trying to draw the adolescent to conversation by explaining the rationale of therapy (item 57) and establishing links between the patient–therapist relationship and significant others (item 100). Negative loading items indicated the adolescent’s reluctance to reveal aggressive feelings (item 84r), avoidance of topics in which he/she was treated unfairly (item 55r), and taking on himself/herself the responsibility of situations (item 34r).

IS 5 accounted for 4.78% of the variance (*α* = 0.82) and was named “Exploratory Psychodynamic Technique (EPT)”. Positive loading items represented exploratory interventions. These included the therapist’s inquiry into the young person’s internal states and affects (item 97) as well as symptoms (item 39), therapist’s elaboration on and clarification of the issues discussed (items 31, 65), facilitation of the patient’s speech (item 3), and expansion on the patient’s views (item 99). The therapist spoke in a clear and understandable fashion (46), projected a nonjudgmental attitude (18), and tried to facilitate the patient’s speech (item 3). Negative loading items showed the therapist’s abstinence, such as refraining from disclosing personal information (item 21r) or his/her emotional responses (item 81r), lack of supportive and behavioral techniques, such as giving explicit guidance (item 27r) or direct reassurance (item 66r), encouraging alternate ways of relating to others (item 85r), and discussing tasks to be performed outside therapy (item 49r).

### 3.2. Descriptive Statistics and Partial Correlations

Table 1 provides the descriptive statistics and the partial correlations between the aggregate IS and BPM-Y Total Problems scores of the patients, controlling for their sex, ages, and problem level scores at baseline (YSR Total Problems). IS 1 correlated significantly with IS 3, IS 4, and IS 5 (*p* = 0.03; *p* = 0.03; *p* = 0.03; consecutively) and IS 2 correlated significantly with IS 5 (*p* = 0.046). None of the other correlations were significant.

### 3.3. MLM Analyses

Examination of the MCMC diagnostics and tests of convergence indicated a “burn-in” of 500 followed by 5000 iterations to be adequate. The ESS for all parameters ranged from 2531 to 4715 (*M* ESS = 3736), which indicated sufficient sample size for all parameter estimations and a good quality estimate. The trace estimates and accuracy diagnostics of model parameters were satisfactory (Supplementary Materials: Figures S1 and S2).

Change over time. The main effect of time on BPM-Y Total Problems indicated a significant linear decrease (*β* = −1.11, *SE* = 0.05, 95% credible interval −0.20 to −0.02, *p* = 0.09).

**Figure 1:**
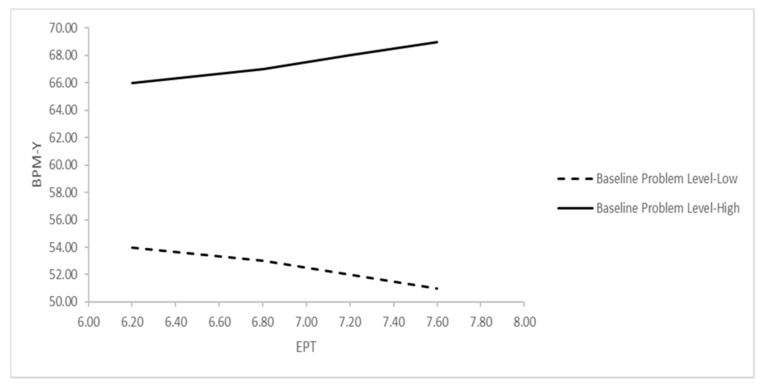
Interaction effect of EPT and YSR Total Problems on prediction of BPM-Y Total Problems. Notes: BPM-Y = Brief Problem Monitor-Youth, EPT = Exploratory Psychodynamic Technique, YSR = Youth Self Report; “Baseline Problem Level–High” indicates 1 *SD* above, and “Baseline Problem Level–Low” indicates 1 *SD* below the mean of pretreatment YSR Total Problems *T*-scores.

Effect of IS on the outcome. The parameters of the second MLM equation are presented in Table 2. No significant direct effect of IS on BPM-Y Total Problems was found (IS 1: *p* = 0.28; IS 2: *p* = 0.48; IS 3: *p* = 0.06; IS 4: *p* = 0.33; IS 5 (EPT): *p* = 0.49). However, a two-way interaction effect between EPT and problem levels at baseline was significant (*p* = 0.037). Figure 1 further clarifies the nature of the interaction, which indicates that greater use of EPT predicted an increase in problem levels for patients who started treatment with higher levels of problems at baseline (i.e., one *SD* above the overall mean), but predicted a decrease in problem levels for those with lower levels of problems at baseline (i.e., one *SD* below the overall mean).

## 4. Discussion

Our first research question concerned the emergent IS in our dataset. As expected, we found an IS associated with the psychodynamic technique (i.e., EPT). This IS represented the different types of interventions employed in psychodynamic psychotherapy, in particular the ones that involve exploration and clarification [36,37]. We found four more IS pertaining to the quality of the therapeutic interaction. These IS signified the typical alliance characteristics that could emerge in the psychodynamic therapy process. The second aim of this study was to investigate the associations between IS and outcome. None of the IS had a direct effect on outcome. EPT only predicted good outcome for patients with less severe problem levels.

IS 1 was named “Negative Therapeutic Alliance”. It bears a resemblance to the factor “Alliance Rupture—Withdrawal” in the study by Di Lorenzo and Maggiolini (2019) [22], but carries more strongly the characteristics of a confrontation rupture rather than a withdrawal rupture [38]. The adolescent is uncommitted to the work of therapy, rejects the therapist’s interventions, refuses to initiate or elaborate upon topics, is provocative, and remains silent. Moreover, the therapeutic bond is weakened in that the adolescent is wary and expresses negative attitudes toward the therapist. Most of the items showed a negative relationship with the “Therapeutic Alliance” factors (which were indicative of a positive alliance) found in studies by Halfon et al. (2020) [23] and Price and Jones (1998) [39].

IS 2 was labeled “Demanding Patient, Accommodating Therapist”. It characterizes one of the central inner conflicts that is definitive of adolescence. On the one hand, the young person is demanding and controlling of the therapeutic relationship, possibly wanting to assert his/her individuality. On the other hand, the patient also resists being autonomous, projects his/her thoughts to the therapist, and wishes to gain the approval of the therapist. Such ambivalence, along with lack of clarity in thought processes, are reminiscent of second individuation [40,41]. The adolescent’s demands and control attempts also fit with the characteristics of a confrontation rupture [38]. The therapist, instead of practicing psychoeducation or taking a strong position, remains thoughtful, neutral, and accommodates the patient if there is difficulty in the relationship. This strategy adheres to the main principles of PDT in adolescents, which aims to form a nonintrusive facilitating environment [42,43]. However, the therapist at times asserts his/her point of view with certainty, possibly a countertransferential response to the patient’s controlling attitude, which may also exacerbate the patient’s sense of being misunderstood. Qualitative studies of adolescent’s experiences of psychotherapy have found that adolescents are especially conscious of their need for autonomy and control within the therapeutic encounter, and this necessitates the practice of an equal relational field [44,45].

IS 3 was named “Emotionally Distant Resistant Patient”. These sessions showed significant withdrawal markers, such as minimal response, avoidance of negative emotions, and denial [38]. IS 3 (similar to IS 1) shares items with the “Alliance Rupture—Withdrawal” factor in the study of Di Lorenzo and Maggiolini (2019) [22] and the third-session cluster describing a disengaged patient in a study by Calderon et al. (2019) [20]. Hence, these types of ruptures seem to be common with adolescents in psychodynamic process. In this IS, the therapist attends to the adolescent’s nonverbal behavior by trying to bring to consciousness unverbalized emotional states and contain them instead of making explicit references to the adolescent’s experience.

IS 4 was termed “Shy Patient, Inviting Therapist”. It provided a profile of a young person who is unable to express anger or object to unfairness. In this respect, the young person is self-deprecating as well as withdrawn (as in IS 3). Such dynamics have been identified with respect to depressive adolescents who tend to turn aggression against themselves and experience self-esteem problems [46]. The therapist’s response shows an effort to engage the young person by explaining the rationale behind therapy, trying to articulate unexpressed somatic sensations, and relating the therapeutic relationship with those in the patient’s life. These techniques have been identified as rupture-resolution strategies, namely providing a rationale for treatment, and focusing on the therapeutic relationship [38]. Moreover, focusing on somatic sensations could be a way to bring to surface the disavowed feelings (i.e., anger).

IS 5 was named EPT. Though not encompassing the interpretive practices of the psychodynamic approach, IS 5 also excludes some supportive practices (e.g., direct reassurance or advice), structured CBT techniques (e.g., discussion of tasks), or more direct techniques (e.g., explaining the meaning of the other’s behaviors). Instead, the therapist conveys nonjudgmental acceptance and focuses on the exploration of feelings, symptoms, and different perspectives while clarifying their meaning and encouraging further elaboration. These fall under exploratory psychodynamic interventions [36,37]. Furthermore, the therapist refrains from divulging any information about his/herself or what he/she is feeling, thereby establishing the way to a transferential relationship. The fact that this IS did not contain interpretative practices may be due to a few reasons. In the largest RCT of PDT for adolescents, the most commonly used techniques were encouraging the patient to experience and express feelings in sessions as well as exploring difficult feelings, encouraging alternative ways to understand experiences, and allowing the patient to initiate the discussion of significant issues [47]. Those techniques are also indicative of an exploratory approach focusing especially on emotions rather than more interpretative practices. For adolescents who have more limited symbolic capacity, verbal interpretations may feel as if they are being accused or asked to take on responsibility for their wrongdoings [46], so such interpretations must be timed and phrased tactfully. Therapists may modify their adherence to certain techniques (i.e., interpretations) depending on the adolescent’s experiences, which is an important component of successful treatments [48].

The second aim of our study was to investigate the associations between IS and the outcome. None of the IS had a direct effect on outcome, suggesting that IS alone are insufficient to account for symptom level changes. However, our findings indicated that EPT predicted a good outcome for patients with less severe baseline problem levels. In contrast, there was an increase in problem levels in the context of EPT for patients with higher baseline problem levels. Some studies with adults and children have revealed similar results [24,25]. The IMPACT study [19] also showed that even though psychosocial treatments for adolescent depression have comparable effects on general and specific psychopathology, a psychoeducational, didactic approach may be indicated for youths with comorbid conduct problems, who have more severe symptoms [49].

### 4.1. Clinical and Research Implications

The IS found in the present study indicate the importance of the therapeutic relationship in PDT for adolescents, as well as the session-level alliance ruptures (confrontation and withdrawal) that can occur over the course of treatment. Ruptures have been studied closely in very few studies with adolescents: further research is needed. Exploring which types of rupture occur and their importance in sessions are crucial given that unaddressed ruptures could lead to premature dissatisfied dropout from PDT by adolescents [50]. Moreover, very few studies have investigated the strategies for rupture resolution with young people [51]. The therapists in our study accommodated the patient if difficult confrontation ruptures emerged, tried to remain thoughtful to provide a nonintrusive environment, focused on the adolescent’s markers of nonverbal emotion, and tried to explain the rationale of therapy if withdrawal markers emerged. Future research should investigate further the types of resolution strategies with adolescents and identify which strategies are more productive.

The final IS associated with EPT and its relationship with the outcome have important clinical implications. PDT, even if encompassing exploratory techniques, could be contraindicated with some adolescents with more severe baseline problems. These adolescents may need more supportive and structured interventions that buttress their ego functions before starting exploratory work, which requires a capacity to tolerate introspection and a certain level of insight. Studies in adults have indicated that the level of insight mediates the associations between interventions and the outcome, but those findings have not been replicated with adolescents [52]. Other studies with adults and children have suggested that the relationship between the psychodynamic technique and outcome depends upon the strength of the therapeutic alliance (e.g., [24,52]). Future studies should investigate whether the therapeutic alliance moderates the association between technique and the outcome in adolescents. Importantly, the APQ is not an explicit measure of technique and includes more global therapist, patient, and relationship characteristics. Future studies should employ a more specific measure of the psychodynamic technique to assess its impact on the outcome.

### 4.2. Study Limitations and Directions for Future Research

This study pointed to empirically derived treatment processes, some of which were found to be prognostic. However, our study had six main limitations. First, treatment was not randomized, so causal inferences were not possible. Second, the study cohort was small, and there were no children with discrete externalizing problems in the cohort. A larger cohort with different diagnoses could reveal whether these findings can be generalized. Moreover, mid/late adolescents (15–17 years) were underrepresented in the dataset (20%). Third, we did not assess other potential mediating variables pertaining to therapist and patient characteristics, which should be assessed in future research. Fourth, the therapists were novice clinicians with limited experience, which may have influenced our results. Fifth, we could assess the outcome only in terms of problem levels. However, the psychodynamic technique may be related to other mechanisms, such as affect regulation, tolerance, and insight, which may have roles in symptom reduction [53]. Moreover, long-term follow-up is necessary because the literature suggests that the effects of psychodynamic treatments increase during follow-up [1]. Finally, collecting information from multiple sources (especially if parents are involved in the process) can provide insight into different aspects of the change from different perspectives.

## 5. Conclusions

We revealed, for the first time in depth, the IS that occur in PDT for adolescents with nonclinical, internalizing, and comorbid internalizing–externalizing problems, and assessed the associations between IS and outcome. Our findings provide preliminary evidence for putative treatment processes in PDT for adolescents and show which treatment aspects may facilitate change. One must pay close attention to the types of alliance ruptures that may occur in sessions and moderate the dose of supportive and exploratory interventions depending on the problem severity of adolescents.

## Figures and Tables

**Table 1:** Partial correlations.

Variables	*M*	*SD*	1	2	3	4	5	6
(1) BPM-Y	60.619	8.472	−					
(2) IS 1	4.101	0.710	−0.033	−				
(3) IS 2	4.460	0.500	−0.111	0.180	−			
(4) IS 3	4.519	0.523	−0.126	0.350 *	0.248	−		
(5) IS 4	4.949	0.550	−0.120	0.346 *	0.026	0.244	−	
(6) IS 5 (EPT)	7.348	0.353	0.149	−0.361 *	−0.322 *	−0.134	−0.251	−

Notes: BPM-Y = Brief Problem Monitor-Youth, IS = Interaction Structure, EPT = Exploratory Psychodynamic Technique, YSR = Youth Self Report, * *p* < 0.05.

**Table 2:** Effect of IS on BPM-Y.

Intercept and Predictors	BPM-Y		
	*B*	*SE*	95% Credible Interval
Intercept (β_00_)	59.798 **	0.789	58.246; 61.352
Sex (β_01_)	−1.176	1.109	−3.337; 1.001
Age (β_02_)	−0.451	0.309	−1.039; 0.167
YSR (β_03_)	0.727 **	0.051	0.627; 0.832
IS 1 (β_10_)	0.402	0.681	−0.935; 1.743
IS 2 (β_20_)	0.040	0.745	−1.443; 1.510
IS 3 (β_30_)	−1.257	0.780	−2.830; 0.251
IS 4 (β_40_)	−0.325	0.736	−1.775; 1.132
IS 5 (EPT) (β_50_)	0.061	1.153	−2.227; 2.334
IS 5 (EPT) × YSR (β_53_)	0.180 *	0.102	−0.016; 0.383

Notes: BPM-Y = Brief Problem Monitor-Youth, IS = Interaction Structures, EPT = Exploratory Psychodynamic Technique, YSR = Youth Self-Report; * *p* < 0.05, ** *p* < 0.01.

## Data Availability

The data presented in this study are available upon request from the corresponding author. The data are not publicly available due to confidentiality and privacy matters.

## Supplementary Materials

The following are available online at https://www.mdpi.com/article/10.3390/ijerph182413007/s1, Supplementary Materials include MCMC diagnostics for all MLM model parameters: Figure S1: MCMC Diagnostics for main parameters (time) of the MLM model assessing change over time in problem levels, Figure S2: MCMC Diagnostics for main the parameters of the MLM model assessing the effect of IS on the outcome.

## Appendix A

**Table A1:** Five-factor solution and item loadings of the APQ items.

**IS 1: Negative Therapeutic Alliance**		
**Item**	**Loading**	**Description**
15	0.681	YP does not initiate or elaborate topics
7	0.669	YP is anxious or tense
44	0.609	YP feels wary or suspicious of the T
30	0.559	YP has difficulty beginning the session
12	0.523	Silences occur during the session
20	0.503	YP is provocative, tests limits of therapy relationship
1	0.496	YP expresses, verbally or nonverbally, negative feelings toward the T
42	0.448	YP rejects T’s comments and observations
23	−0.471	YP is curious about the thoughts, feelings, or behavior of others
24	−0.478	YP demonstrates capacity to link mental states with action or behavior
28	−0.482	YP communicates a sense of agency
74	−0.575	Humor is used
13	−0.627	YP is animated or excited
95	−0.654	YP feels helped by the therapy
72	−0.684	YP demonstrates lively engagement with thoughts and ideas
73	−0.785	YP is committed to the work of therapy
**IS 2: Demanding Patient, Accommodating Therapist**		
**Item**	**Loading**	**Description**
87	0.757	YP is controlling of the interaction with the T
83	0.669	YP is demanding
78	0.602	YP seeks T’s approval, affection, or sympathy
14	0.528	YP does not feel understood by T
93	0.501	T refrains from taking position in relation to YP’s thoughts or behavior
89	0.461	T makes definite statements about what is going on in the YP’s mind
47	0.421	When the interaction with YP is difficult, T accommodates in an effort to improve relations
51	0.411	YP attributes own characteristics or feelings to T
37	0.401	T remains thoughtful when faced with YP’s strong affect or impulses
29	−0.408	YP talks about wanting to be separate or autonomous from others
80	−0.439	T presents an experience or event from a different perspective
54	−0.458	YP is clear and organized in self-expression
33	−0.609	T adopts a psychoeducational stance
**IS 3: Emotionally Distant Resistant Patient**		
**Item**	**Loading**	**Description**
58	0.565	YP resists T’s attempts to explore thoughts, reactions, or motivations related to problems
53	0.507	YP discusses experiences as if distant from his feelings
10	0.500	YP displays feelings of irritability
67	0.495	YP finds it difficult to concentrate or maintain attention during the session
2	0.426	T draws attention to YP’s nonverbal behavior
22	−0.404	YP expresses feelings of remorse
59	−0.408	YP feels inadequate and inferior
32	−0.459	YP achieves a new understanding
6	−0.487	YP describes emotional qualities of the interactions with significant others
94	−0.504	YP feels sad or depressed
41	−0.514	YP feels rejected or abandoned
9	−0.525	T works with YP to try to make sense of experience
26	−0.660	YP experiences or expresses troublesome (painful) effects
8	−0.722	YP expresses feelings of vulnerability
**IS 4: Inexpressive Patient, Inviting Therapist**		
**Item**	**Loading**	**Description**
77	0.501	T encourages YP to attend to somatic feelings or sensations
61	0.496	YP feels shy or self-conscious
57	0.430	T explains rationale behind technique or approach to treatment
100	0.405	T draws connections between the therapeutic relationship and other relationships
52	−0.448	YP has difficulty with ending of sessions
34	−0.465	YP blames others or external forces for difficulties
88	−0.539	YP fluctuates between strong emotional states during the session
55	−0.641	YP feels unfairly treated
84	−0.770	YP expresses angry or aggressive feelings
**IS 5: Exploratory Psychodynamic Technique (EPT)**		
**Item**	**Loading**	**Description**
65	0.632	T restates or rephrases YP’s communication in order to clarify its meaning
99	0.579	T raises questions about YP’s view
3	0.545	T’s remarks are aimed at facilitating YP’s speech
97	0.533	T encourages reflection on internal states and affects
18	0.511	T conveys a sense of nonjudgmental acceptance
39	0.468	T encourages YP to reflect on symptoms
46	0.452	T communicates with YP in a clear, coherent style
31	0.407	T asks for more information or elaboration
66	−0.408	T is directly reassuring
76	−0.413	T explicitly reflects on own behavior, words or feelings
21	−0.417	T self-discloses
85	−0.484	T encourages YP to try new ways of behaving with others
81	−0.575	T reveals emotional responses
43	−0.618	T suggests the meaning of others’ behavior
49	−0.618	There is discussion of specific activities or tasks for the YP to attempt outside of session
27	−0.626	T offers explicit advice and guidance

Notes: APQ = Adolescent Psychotherapy Q-Set, IS = Interaction Structures, T = Therapist, YP = Young Person.
