# Supplementary material for: Interaction Structures in Psychodynamic Psychotherapy for Adolescents
Source: Int J Environ Res Public Health. 2021 Dec 9;18(24):13007. doi: 10.3390/ijerph182413007 (PMC8701824; doi:10.3390/ijerph182413007)

Figure S1: MCMC Diagnostics for main parameters (time) of the MLM model assessing change over time in problem behaviors

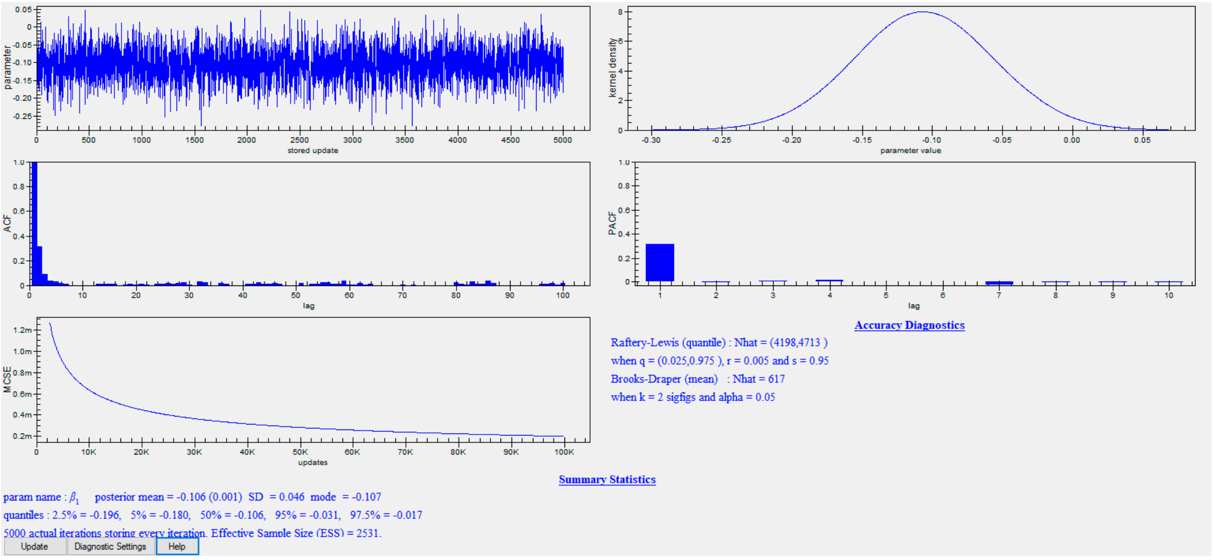

Supplement: Supplementary file 1 [file ijerph-18-13007-s001.zip › ijerph-1431259-Supplementary Figure S1.pdf]
