# Supplementary material for: Interaction Structures in Psychodynamic Psychotherapy for Adolescents
Source: Int J Environ Res Public Health. 2021 Dec 9;18(24):13007. doi: 10.3390/ijerph182413007 (PMC8701824; doi:10.3390/ijerph182413007)
Supplement: Supplementary file 1 [file ijerph-18-13007-s001.zip › ijerph-1431259-Supplementary Figure S2.pdf]

Figure S2: MCMC Diagnostics for main the parameters of the MLM model assessing the effect of IS on the outcome

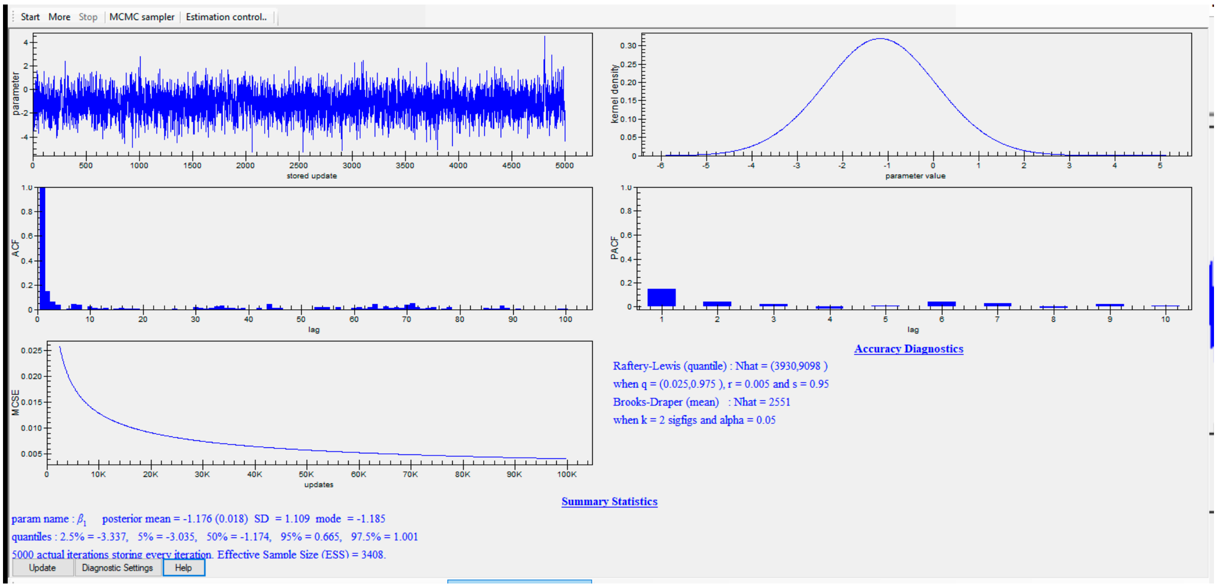

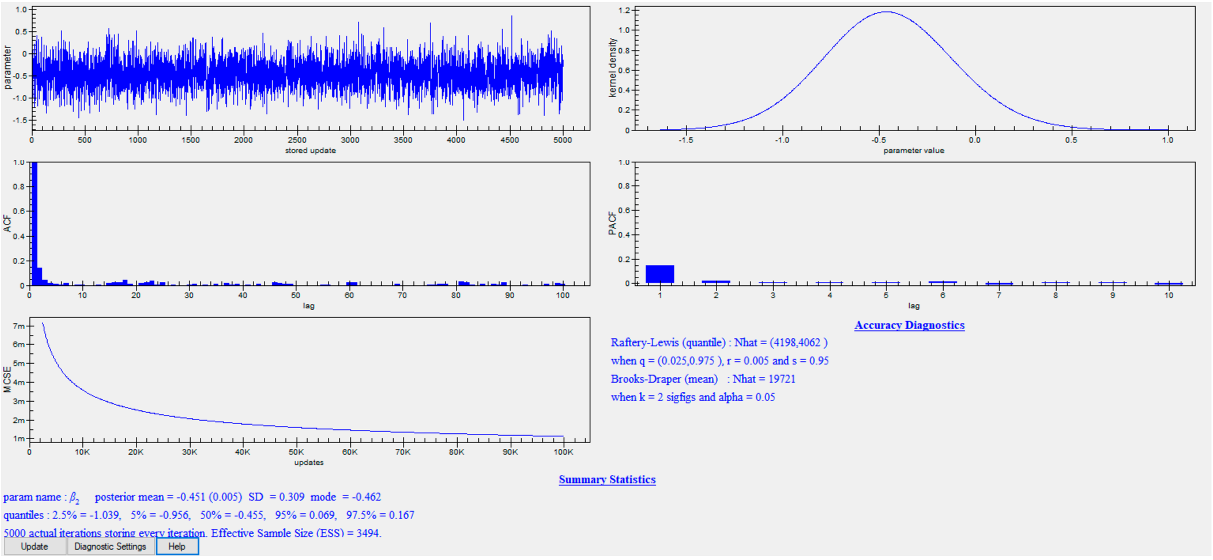

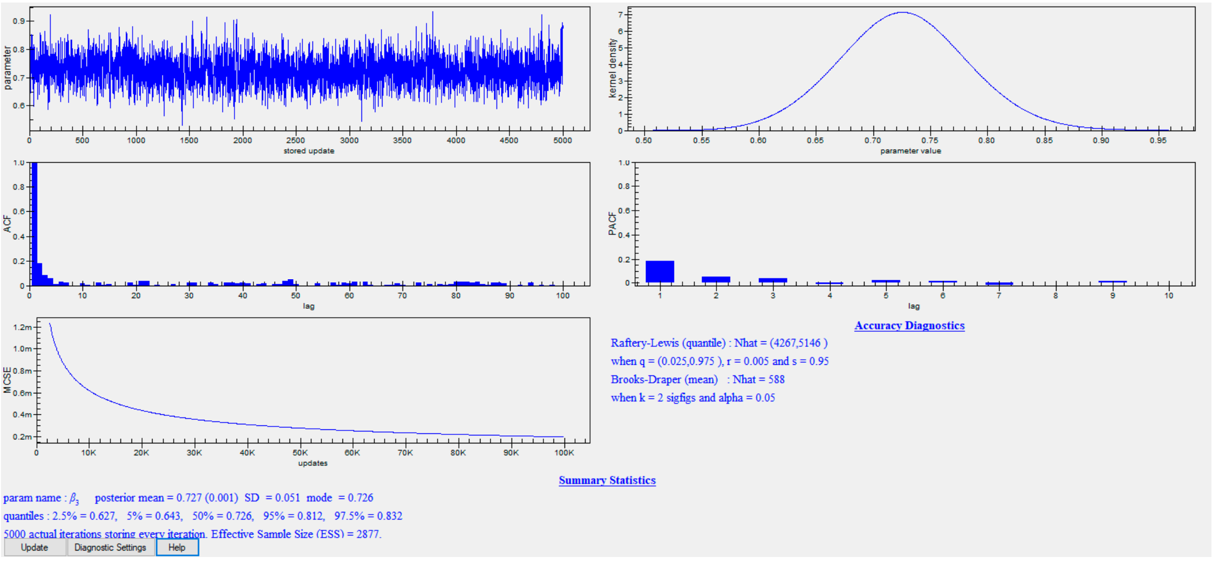

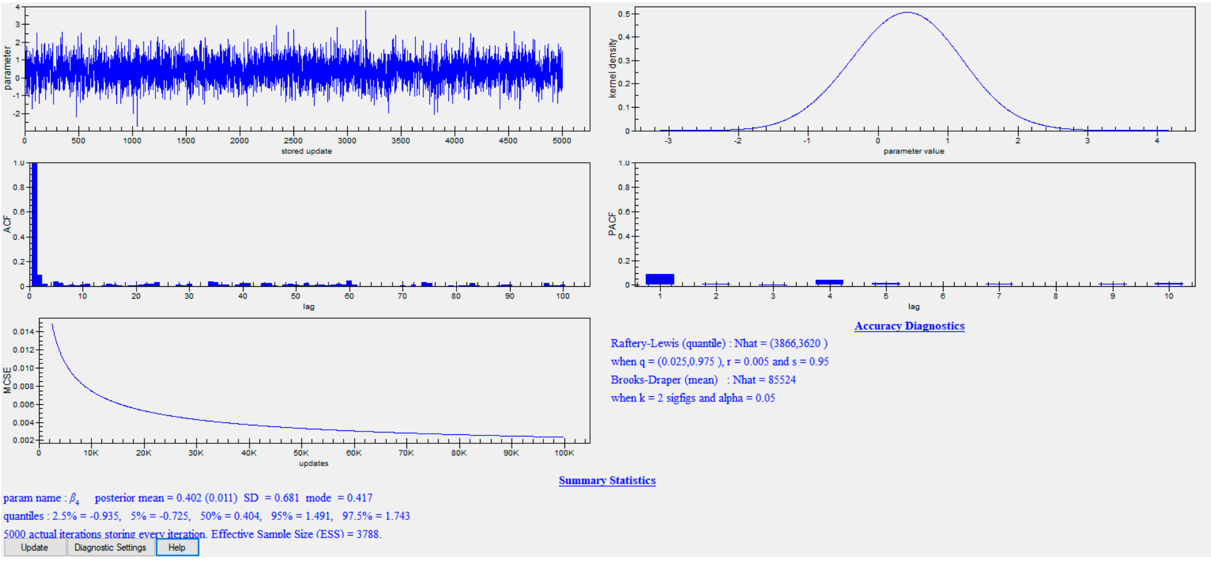
Accuracy Diagnostics
Raftery-Lewis (quantile) : Nhat = (3866,3620)
when q = (0.025,0.975), r = 0.005 and s = 0.95
Brooks-Draper (mean) : Nhat = 85524
when k = 2 sigfigs and alpha = 0.05
Summary Statistics
param name : β4 posterior mean = 0.402 (0.011) SD = 0.681 mode = 0.417
quantiles : 2.5% = -0.935, 5% = -0.725, 50% = 0.404, 95% = 1.491, 97.5% = 1.743
5000 actual iterations storing every iteration. Effective Sample Size (ESS) = 3788.
Update
Diagnostic Settings
Help

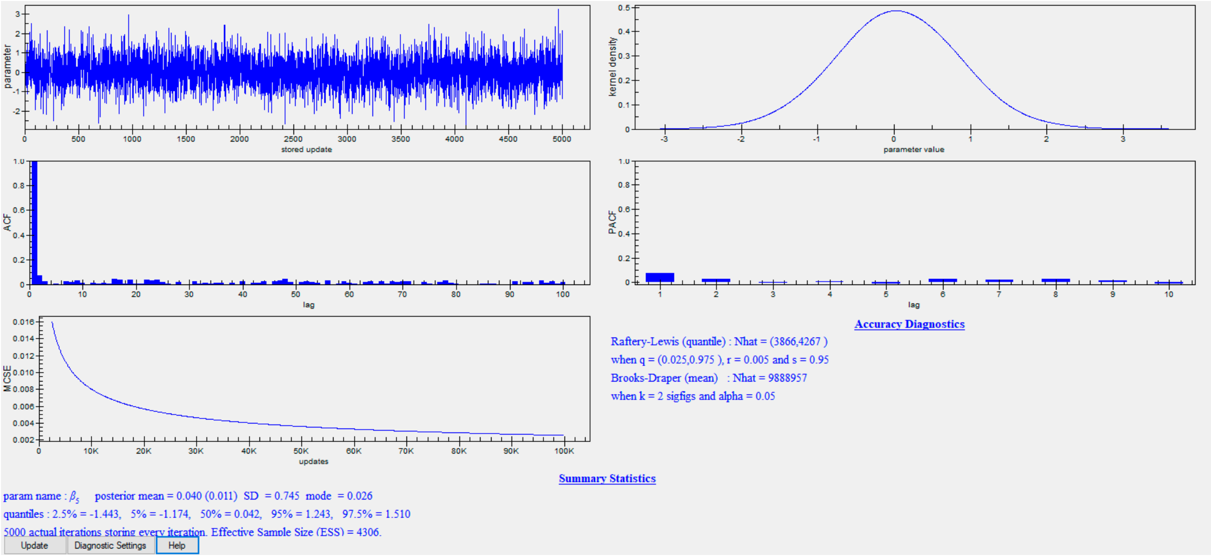
Accuracy Diagnostics
Raftery-Lewis (quantile) : Nhat = (3866,4267)
when q = (0.025,0.975), r = 0.005 and s = 0.95
Brooks-Draper (mean) : Nhat = 9888957
when k = 2 sigfigs and alpha = 0.05
Summary Statistics
param name : β5 posterior mean = 0.040 (0.011) SD = 0.745 mode = 0.026
quantiles : 2.5% = -1.443, 5% = -1.174, 50% = 0.042, 95% = 1.243, 97.5% = 1.510
5000 actual iterations storing every iteration. Effective Sample Size (ESS) = 4306.
Update
Diagnostic Settings
Help

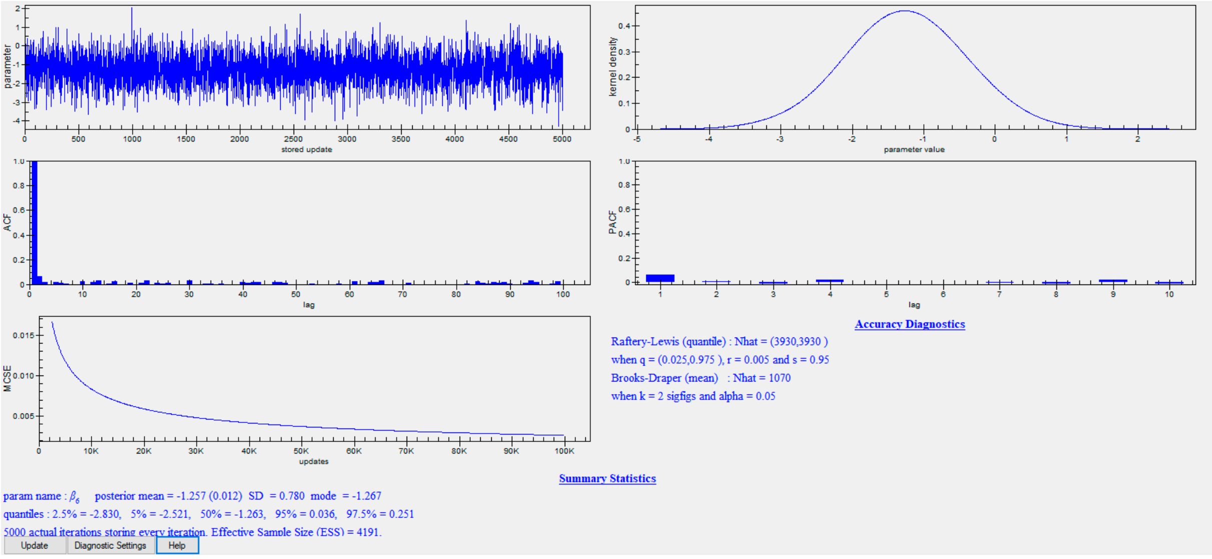
Accuracy Diagnostics
Raftery-Lewis (quantile) : Nhat = (3930,3930)
when q = (0.025,0.975), r = 0.005 and s = 0.95
Brooks-Draper (mean) : Nhat = 1070
when k = 2 sigfigs and alpha = 0.05
Summary Statistics
param name : β6 posterior mean = -1.257 (0.012) SD = 0.780 mode = -1.267
quantiles : 2.5% = -2.830, 5% = -2.521, 50% = -1.263, 95% = 0.036, 97.5% = 0.251
5000 actual iterations storing every iteration. Effective Sample Size (ESS) = 4191.
Update
Diagnostic Settings
Help

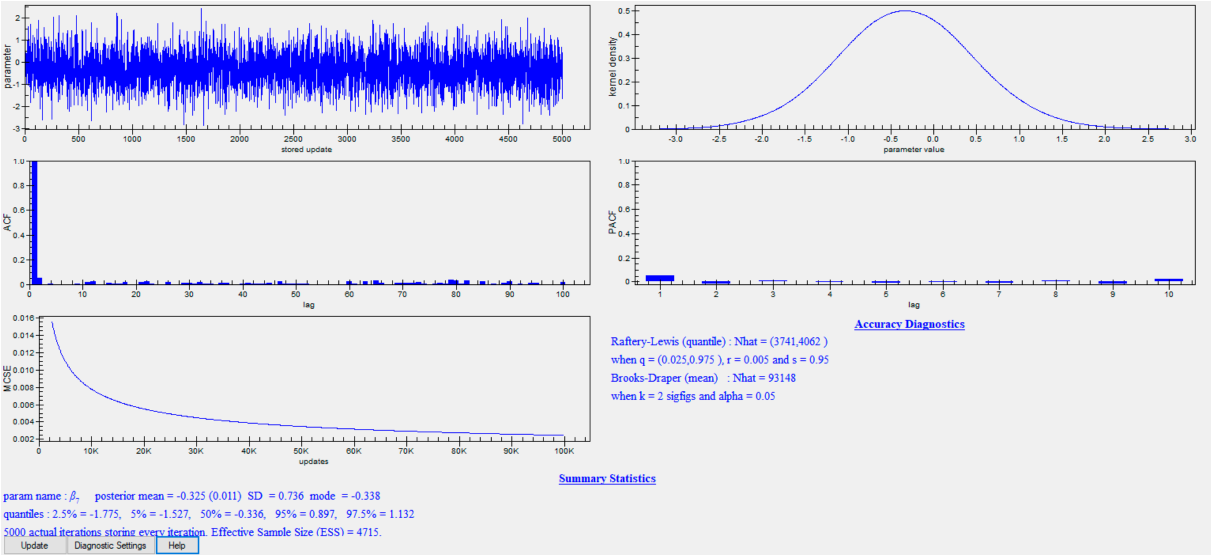
parameter
stored update
kernel density
parameter value
ACF
lag
PACF
lag
MCSE
updates
Accuracy Diagnostics
Raftery-Lewis (quantile) : Nhat = (3741,4062)
when q = (0.025,0.975), r = 0.005 and s = 0.95
Brooks-Draper (mean) : Nhat = 93148
when k = 2 sigfigs and alpha = 0.05
Summary Statistics
param name : β_7 posterior mean = -0.325 (0.011) SD = 0.736 mode = -0.338
quantiles : 2.5% = -1.775, 5% = -1.527, 50% = -0.336, 95% = 0.897, 97.5% = 1.132
5000 actual iterations storing every iteration. Effective Sample Size (ESS) = 4715.
Update
Diagnostic Settings
Help

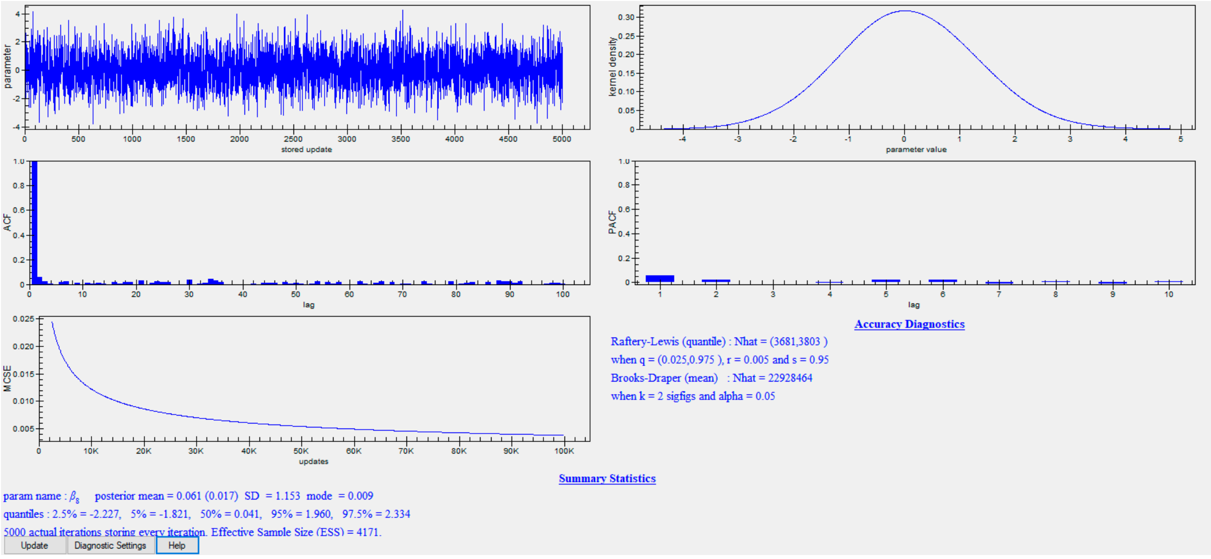
parameter
stored update
kernel density
parameter value
ACF
lag
PACF
lag
MCSE
updates
Accuracy Diagnostics
Raftery-Lewis (quantile) : Nhat = (3681,3803)
when q = (0.025,0.975), r = 0.005 and s = 0.95
Brooks-Draper (mean) : Nhat = 22928464
when k = 2 sigfigs and alpha = 0.05
Summary Statistics
param name : β_8 posterior mean = 0.061 (0.017) SD = 1.153 mode = 0.009
quantiles : 2.5% = -2.227, 5% = -1.821, 50% = 0.041, 95% = 1.960, 97.5% = 2.334
5000 actual iterations storing every iteration. Effective Sample Size (ESS) = 4171.
Update
Diagnostic Settings
Help

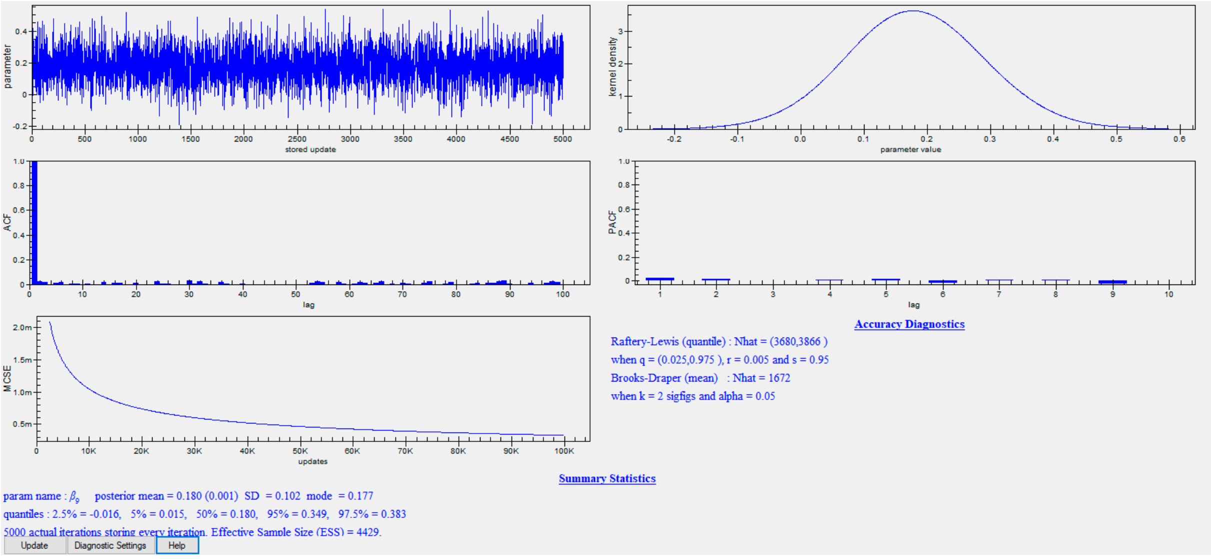
parameter
stored update
kernel density
parameter value
ACF
lag
PACF
lag
MCSE
updates
Accuracy Diagnostics
Raftery-Lewis (quantile) : Nhat = (3680,3866)
when q = (0.025,0.975), r = 0.005 and s = 0.95
Brooks-Draper (mean) : Nhat = 1672
when k = 2 sigfigs and alpha = 0.05
Summary Statistics
param name : β_9 posterior mean = 0.180 (0.001) SD = 0.102 mode = 0.177
quantiles : 2.5% = -0.016, 5% = 0.015, 50% = 0.180, 95% = 0.349, 97.5% = 0.383
5000 actual iterations storing every iteration. Effective Sample Size (ESS) = 4429.
Update
Diagnostic Settings
Help
